# Radio-induced malignancies after breast cancer postoperative radiotherapy in patients with Li-Fraumeni syndrome

**DOI:** 10.1186/1748-717X-5-104

**Published:** 2010-11-08

**Authors:** Steve Heymann, Suzette Delaloge, Arslane Rahal, Olivier Caron, Thierry Frebourg, Lise Barreau, Corinne Pachet, Marie-Christine Mathieu, Hugo Marsiglia, Céline Bourgier

**Affiliations:** 1 Department of Radiation Oncology, Institut Gustave Roussy, Villejuif, France; 2 Department of Breast Oncology, Institut Gustave Roussy, Villejuif, France; 3 Department of Genetics Counseling, Institut Gustave Roussy, Villejuif, France; 4 Genetic Department, Academic Hospital, Rouen, France; 5 Department of Breast Surgery, Institut Gustave Roussy, Villejuif, France; 6 Department of Pathology, Institut Gustave Roussy, Villejuif, France; 7 University of Florence, Italy

## Abstract

**Background:**

There are no specific recommendations for the management of breast cancer patients with germ-line p53 mutations, an exceptional genetic condition, particularly regarding postoperative radiotherapy. Preclinical data suggested that p53 mutations conferred enhanced radiosensitivity *in vitro *and *in vivo *and the few clinical observations showed that Li-Fraumeni families were at a higher risk of secondary radio-induced malignancies.

**Methods:**

We reviewed a cohort of patients with germ-line p53 mutations who had been treated for breast cancer as the first tumor event. We assessed their outcome and the incidence of secondary radio-induced malignancies.

**Results:**

Among 47 documented Li-Fraumeni families treated from 1997 to 2007 at the Institut Gustave Roussy, 8 patients had been diagnosed with breast cancer as the first tumor event. Three patients had undergone conservative breast surgery followed by postoperative radiotherapy and five patients had undergone a mastectomy (3 with postoperative radiotherapy). Thus, 6/8 patients had received postoperative radiotherapy. Median follow-up was 6 years. Median age at the diagnosis of the primary breast cancer was 30 years. The histological characteristics were as follows: intraductal carcinoma *in situ *(n = 3), invasive ductal carcinoma (n = 4) and a phyllodes tumor (n = 1). Among the 6 patients who had received adjuvant radiotherapy, the following events had occurred: 3 ipsilateral breast recurrences, 3 contralateral breast cancers, 2 radio-induced cancers, and 3 new primaries (1 of which was an in-field thyroid cancer with atypical histology). In contrast, only one event had occurred (a contralateral breast cancer) among patients who had not received radiation therapy.

**Conclusions:**

These observations could argue in favor of bilateral mastectomy and the avoidance of radiotherapy.

## Background

Li-Fraumeni syndrome (LFS) is a rare disorder that considerably increases the risk of developing several types of cancer, particularly in children and young adults. The first observations were described by Li and Fraumeni in 1969 [1]. LFS is inherited in an autosomal dominant pattern with the frequent occurrence of soft tissue/bone sarcoma, breast cancer, leukemia, brain tumors and other cancers (melanoma, colon cancer, pancreatic cancer, adrenocortical carcinoma) [1,2]. Since then, several reports of affected families have contributed to a more precise definition of the Li Fraumeni syndrome [3].

Germ-line *TP53 *gene mutations are mainly reported in LFS and approximately 250 distinct germ-line *TP53 *mutations have been described in the literature [4]. A *TP53 *mutation database has been established http://www-p53.iarc.fr/[5]. Mutations in the *CHEK2 *gene have also been reported in a few LFS and Li Fraumeni-like syndrome (LFL) families [6-8]. Wild-type p53 was identified as the first tumor suppressor gene. It is at the crossroads of the network of signaling pathways involved in the elimination and inhibition of abnormal cell proliferation designed to prevent neoplastic development [9,10]. Many transcriptional targets of wild-type p53 have been implicated: (i) in cell cycle inhibition by maintaining cells in the G2 cell cycle arrest, for example, the cyclin-dependent kinase inhibitor p21^Waf1^, 14-3-3sigma (σ); (ii) in the regulation of apoptosis through the induction of pro-apoptotic proteins such as Bax, Apaf 1, PUMA, p53AIP1, PIDD and NOXA; (iii) in DNA repair; (iv) in angiogenesis and in metastasis inhibition [11-13]. p53 gene inactivation is essentially due to small mutations which lead to either the expression of a mutant protein (90% of cases) or the absence of protein expression (10% of cases). Here, we attempted to assess the incidence of radio-induced malignancies in a prospective cohort of families with germ-line p53 mutations, focusing on breast cancer occurring as the first malignancy.

## Methods

We conducted a search of the genetic screening database at the Institut Gustave Roussy (*Villejuif) *for "female AND breast cancer AND mutation of TP53" from 1997 to 2007. Clinical, pathological, and treatment characteristics were assessed and the analysis was performed in February 2010. A loco-regional relapse was defined as an ipsilateral relapse in either the breast or lymph node-bearing areas (axillary, internal mammary, supra-clavicular) or both occurring since the date of the diagnosis. Contralateral breast cancer was either ductal carcinoma *in situ *(DCIS) or invasive carcinoma. Distant disease was defined as breast carcinoma recurrences that were not in the contralateral breast nor in loco-regional areas. Second primaries were recorded in the database.

## Results

Among 47 families with either LFS or LFL syndrome, eight patients were recorded as having a breast cancer as the first malignancy. The median follow-up was 6 years [2-13]. Median age was 30 years [22-48]. Among those 8 patients, 6 had received loco-regional radiation therapy. After a median follow-up of 6 years since the initial diagnosis [2-13], 3 ipsilateral breast relapses and 4 contralateral breast cancers had occurred and 2 radio-induced cancers (one chest wall angiosarcoma and one breast histiocytofibrosarcoma). One papillary thyroid carcinoma had also developed inside the radiation field, which was considered as a new primary rather than a radio-induced malignancy because of the two years of latency. Two other primaries had also occurred: a buttock liposarcoma and an ethmoidal leiomyosarcoma. Two patients had developed metastases from the primary breast carcinoma and one patient had died of metastatic disease.

### Patient 1

A 27 year-old woman with a familial history of LFS had presented with a 35 mm DCIS of the left breast that had been treated by a radical mastectomy and axillary clearance in 1999. She had no evidence of a relapse.

### Patient 2

A 32 year-old woman with a familial history of LFS had presented with a right breast cancer (Scarff and Bloom and Richardson (SBR) grade 1 invasive ductal carcinoma (IDC), pT1N0, ER+, PR+, HER2-) that had been treated by a radical mastectomy and Tamoxifen in 2007. One year after the initiation of Tamoxifen, a contralateral breast cancer (CBC) had occurred (DCIS) that had been treated by a radical mastectomy.

### Patient 3

A 22 year-old woman with a familial history of multiple breast cancers had presented in 2005 with an IDC of the right breast (cT2N1, ER+, PR+, HER2+) that had been treated with neo-adjuvant chemotherapy and traztuzumab. A radical mastectomy and an axillary dissection(1N+/25) had been performed followed by loco-regional radiotherapy to the chest wall, internal mammary and supraclavicular nodes, endocrine therapy and a prophylactic contralateral mastectomy.

### Patient 4

A 32 year-old woman without any familial history of cancer had presented with an IDC of the right breast (SBR grade 2 T1N1 ER+, PR+, Her2+). She had been treated by a radical mastectomy followed by chemotherapy with traztuzumab, loco-regional irradiation (chest wall, internal mammary and supraclavicular nodes) and Tamoxifen. Before completion of traztuzumab, i.e. 8 months after completion of radiotherapy, a CBC had been diagnosed (left axillary IDC, ER+, PR+, HER2+) that had been treated by a lumpectomy including a sentinel lymph node biopsy and chemotherapy with traztuzumab. The p53 mutation had been diagnosed during chemotherapy. Postoperative radiotherapy had therefore been cancelled and replaced by a mastectomy.

### Patient 5

A 22 year-old woman without any familial history of cancer had presented with a right breast phyllodes tumor in 1997. Conservative breast surgery had been performed followed by adjuvant radiotherapy delivered to the whole breast. In 2001, she had developed a buttock liposarcoma and then a CBC (SBR grade 2 IDC) in 2004 that had been treated by conservative surgery followed by radiotherapy to the breast, internal mammary and supraclavicular nodes. An ipsilateral breast cancer (IBC) had occurred in 2008, ("in-field relapse": a 50 mm, ER-, PR-, Her2+ mucinous carcinoma ). It had been treated by a radical mastectomy and with traztuzumab. Due to the occurrence of multiple malignancies at a very young age in this patient, she had received genetic counseling and a p53 mutation had been diagnosed. At the time of the analysis (Feb. 2010), she developed an ipsilateral chest wall angiosarcoma which is currently being treated with chemotherapy.

### Patient 6

A 29 year-old woman with a familial history of multiple cancers had undergone conservative surgery of the right breast for an IDC (SBR grade 2 T1N1, ER+, PR+, HER2-) in 1998. She had received adjuvant chemotherapy and radiotherapy. An ipsilateral, multicentric breast recurrence (IDC) had developed 10 years later (an in-field relapse of the same histologic type) and had been treated by a radical mastectomy and endocrine therapy. A TP53 mutation had been diagnosed in 2008. At the time of the analysis (Feb. 2010), a contralateral axillary recurrence was diagnosed and treated with chemotherapy.

### Patient 7

A 48 year-old female had presented in 2005 with a right breast cancer (IDC) with axillary lymph node involvement and a concomitant grade 2 malignant histiocytofibroma of the left thigh measuring 8 cm. She had a familial history of cancer (2 brothers with rhabdomyosarcoma, and cancers in both parents). She had received five cycles of adriamycin and ifosfamide (AI), 9 cycles of weekly paclitaxel and had undergone a mastectomy with axillary clearance for the IDC (SBR grade 3 ER+, PR+, HER2-) measuring 120 mm with multiple vascular involvement (VI) and 9N+/16. She had received radiotherapy to the chest wall, internal mammary and supraclavicular nodes and endocrine therapy. She had undergone surgery for the malignant histiocytofibroma of the thigh after the 5 cycles of AI.

In August 2007, she had undergone a thyroidectomy and bilateral neck and superior mediastinal lymph node dissection for a papillary carcinoma with VI, 10N+, followed by radioactive iodine therapy. In April 2008, she had developed a liver metastasis and had been treated with 3 lines of chemotherapy. She had progressive disease at the time of the analysis (Feb 2010).

### Patient 8

A 39 year-old female diagnosed with a DCIS of the left breast had undergone a lumpectomy and had received postoperative radiotherapy and tamoxifen. In 2004, she had developed a local relapse that had been treated by a mastectomy and axillary clearance. Two tumors had been discovered: one grade 2 histiocytofibrosarcoma and 6N+ exhibiting IDC (ER+, PR+, HER2-). She had received adjuvant chemotherapy, radiotherapy to the chest wall, internal mammary and supraclavicular nodes and then endocrine therapy. In 2006, she had developed a grade 2 ethmoidal leiomyosarcoma that had been treated by surgery and radiotherapy. In December 2006, she had presented with a left infracapsular mass which had been diagnosed as metastasis from IDC and had been treated with chemotherapy. She had developed cerebral metastasis in September 2007 and pleural metastasis in December of the same year. She had died at the end of 2008 of disease progression. Her 18 year-old daughter has 2 sarcomas.

## Genomic analysis

### TP53 analysis

The 11 exons of TP53 and intron-exon boundaries were thoroughly analyzed by direct sequencing after genomic DNA amplification. Genomic rearrangements were sought by Quantitative Multiplex Polymerase chain reaction of Short Fragments (QMPSF), as described elsewhere [14].

We screened the mutations on the IARC website http://www-p53.iarc.fr. Table 1 lists the type of germ-line p53 mutation for each patient. The majority of the mutations were missense mutations resulting in abnormal protein function. Patients 1 and 8 had a splicing mutation. The splicing mutation in patient 1 has already been described as a germ-line mutation in 8 LFS families and the mutation in patient 8, which induces buried DNA-binding function, has already been described in 2 LFS families.

**Table 1:** Patient characteristics, outcome and genetic information

	1	2	3	4	5	6	7	8
Age	27	32	22	32	22	29	48	39

Histology	DCIS	IDC and DCIS	IDC	IDC	Phyllodes sarcoma	IDC	IDC	DCIS

Grade	NA	1	NA	2	NA	2	3	NA

Hormonal receptor	UN	pos	pos	pos	NA	pos	pos	pos

HER2 overexpression	NA	neg	pos	pos	NA	neg	neg	NA

TNM	TisN0M0	T1N0M0	T2N1M0	T1N1M0	TxN0M0	T1N1M0	T4N1M0	TisN0M0

Adjuvant Radiotherapy	No	No	Yes	Yes	Yes	Yes	Yes	Yes

Local relapse	No	No	No	No	Yes	Yes	No	Yes

Contralateral breast cancer	No	Yes	No	Yes	Yes	Yes	No	No

Radio induced tumors	No	No	No	No	Yes	No	*	Yes

New primary outside RT field	No	No	No	No	Yes	No	No	Yes

Codon Mutation	c.375G > C exon 4 splice site	c.844C > T exon 8 missense	c.742C > T exon 7 missense	c.467G > A exon 5 missense	c.724T > C exon 7 missense	c.542G > A exon 5 missense	c.524G > A exon 5 missense	c.673-2A > G intron 6 splice site

DCIS: ductal carcinoma in situ; IDC: invasive ductal carcinoma; UN unknown; NA: non applicable; * in field tumor with atypical histology

## Discussion

To our knowledge, this is the first report on breast cancer as the first tumor in LFS, without any previous cytotoxic therapy. A large retrospective cohort study assessed the outcomes of long-term survivors after cancer treatments in childhood. The results were alarming because they suggested that chemotherapy and ionizing radiation exposure increased the incidence of second malignancies. More specifically, radiation exposure among TP53 mutation carriers seemed to increase second cancers [15]. Other small cohort studies have suggested a similar outcome [16-19].

No specific clinical or histological feature of breast cancer occurring as a first event has been described in other series. A young age is commonly associated with an aggressive breast cancer phenotype [20,21]. Furthermore, a young age implied breast cancer mutations, such as BRCA mutations. In BRCA1 mutation carriers, breast cancers mostly exhibited a basal-like molecular phenotype [22].

Besides the histological characteristics of breast cancers associated with a young age, a young age has also been reported to be a poor prognostic factor for distant metastases [23,24]. Nonetheless, in the present study with a median follow-up of 6 years, only 2/8 patients had developed distant metastases. Indeed, our patients had mostly developed either local recurrences or contralateral breast cancer.

In an overall population of patients treated for a breast cancer, the risk of loco-regional relapse after breast surgery and postoperative radiotherapy is commonly reported to be 1% per year. A young age is the main prognostic factor for loco-regional relapses with a first peak before the first 2-3 years after the completion of treatment followed by a decreasing risk over time [20]. Even though the cohort under study was small, an ipsilateral breast relapse ("in-field relapse") had occurred in 3/8 patients (in 2, ten years after the initial diagnosis). In addition, CBC had occurred in 4/8 patients but one had undergone a prophylactic contralateral mastectomy.

Radio-induced cancers are usually a very rare event arising 10 years after irradiation with an incidence of less than 2 ‰ [25]. In the present cohort of LFS, a chest wall angiosarcoma, a malignant histiocytofibroma and a papillary thyroid carcinoma had developed inside the irradiated volumes in 3/8 patients.

Experimental data highlighted the role of ionizing radiation stress in human cells harboring heterozygous germ-line p53 mutations, leading to a defective cell cycle arrest in G1/S and/or a lesser apoptotic response of lymphocytes [26]. All these cellular features may promote radiosensitization and thus carcinogenesis [26]. In addition to these *in vitro *results, *in vivo *studies showed that ionizing radiation accelerated the emergence of solid tumors in Trp53 heterozygous null mice [27]. To reinforce experimental data, a few, albeit, very small retrospective cohort studies have reported a higher risk of developing a radiation-induced malignancy among *TP53 *mutation carriers [16-19].

The events described here are probably the result of the sum of the effects of the genetic background on both the risk of new primaries, especially within the breast, and the risk of radiation-induced carcinogenesis. Recent data highlighted the importance of a familial history of cancer or multiple primary tumours (6/8 patients in our cohort) [2,28]. Thus, we strongly believe that patients with early onset breast cancer should be tested for *TP53 *mutation according to updated Chompret criteria [28].

## Conclusion

If a germ-line mutation is detected, we recommend that it be taken into account for decision-making concerning local treatment: 1. Adjuvant radiation therapy for localized breast cancer should be extensively discussed and prohibited whenever the risk/benefit ratio is doubtful. 2. Both a mastectomy of the cancer-bearing breast and a contralateral prophylactic mastectomy (with immediate reconstruction, as frequently as possible) should be advised and discussed with the patient, as is the case for BRCA1/2 mutation carriers, with the additional advantage of potentially avoiding radiation therapy if conservative treatment is avoided.

## List of abbreviations

LFS: Li-Fraumeni syndrome; LFL: Li-Fraumeni-like syndrome; IDC: invasive ductal carcinoma; DCIS: ductal carcinoma in situ; SBR: Scarff Bloom Richardson; ER: estrogen receptor; PR: progesterone receptor; CBC: contralateral breast cancer; IBC: ipsilateral breast cancer; AI: adriamycin ifosfamide; VI: vascular involvement; QMPSF: Quantitative Multiplex Polymerase chain reaction of Short Fragments.

## Competing interests

The authors declare that they have no competing interests.

## Authors' contributions

SH, SD, and AR reviewed the medical files. OC and TF carried out the molecular genetic studies. SH, SD, CB, HM drafted the manuscript. CB and SD: conception, design. MCM, LB and CP participated in the design of the study. All authors read and approved the final manuscript.

## References

[B1] Li FP Fraumeni JF Jr Soft-tissue sarcomas, breast cancer, and other neoplasms. A familial syndrome? Ann Intern Med 1969 71 747 752 5360287 10.7326/0003-4819-71-4-747

[B2] Gonzalez KD Noltner KA Buzin CH Beyond Li Fraumeni Syndrome: clinical characteristics of families with p53 germline mutations J Clin Oncol 2009 27 1250 1256 10.1200/JCO.2008.16.6959 19204208

[B3] Li FP Fraumeni JF Jr Mulvihill JJ A cancer family syndrome in twenty-four kindreds Cancer Res 1988 48 5358 5362 3409256

[B4] Varley JM Germline TP53 mutations and Li-Fraumeni syndrome Hum Mutat 2003 21 313 320 10.1002/humu.10185 12619118

[B5] Olivier M Goldgar DE Sodha N Li-Fraumeni and related syndromes: correlation between tumor type, family structure, and TP53 genotype Cancer Res 2003 63 6643 6650 14583457

[B6] Lee SB Kim SH Bell DW Destabilization of CHK2 by a missense mutation associated with Li-Fraumeni Syndrome Cancer Res 2001 61 8062 8067 11719428

[B7] Varley J TP53, hChk2, and the Li-Fraumeni syndrome Methods Mol Biol 2003 222 117 129 12710683 10.1385/1-59259-328-3:117

[B8] Varley J Haber DA Familial breast cancer and the hCHK2 1100delC mutation: assessing cancer risk Breast Cancer Res 2003 5 123 125 10.1186/bcr582 12793891 PMC164998

[B9] Vogelstein B Lane D Levine AJ Surfing the p53 network Nature 2000 408 307 310 10.1038/35042675 11099028

[B10] Vousden KH Activation of the p53 tumor suppressor protein Biochim Biophys Acta 2002 1602 47 59 11960694 10.1016/s0304-419x(02)00035-5

[B11] Gasco M Shami S Crook T The p53 pathway in breast cancer Breast Cancer Res 2002 4 70 76 10.1186/bcr426 11879567 PMC138723

[B12] Wang QE Zhu Q Wani MA Tumor suppressor p53 dependent recruitment of nucleotide excision repair factors XPC and TFIIH to DNA damage DNA Repair (Amst) 2003 2 483 499 10.1016/S1568-7864(03)00002-8 12713809

[B13] Fei P El-Deiry WS P53 and radiation responses Oncogene 2003 22 5774 5783 10.1038/sj.onc.1206677 12947385

[B14] Bougeard G Brugieres L Chompret A Screening for TP53 rearrangements in families with the Li-Fraumeni syndrome reveals a complete deletion of the TP53 gene Oncogene 2003 22 840 846 10.1038/sj.onc.1206155 12584563

[B15] Kony SJ de Vathaire F Chompret A Radiation and genetic factors in the risk of second malignant neoplasms after a first cancer in childhood Lancet 1997 350 91 95 10.1016/S0140-6736(97)01116-1 9228960

[B16] Hisada M Garber JE Fung CY Multiple primary cancers in families with Li-Fraumeni syndrome J Natl Cancer Inst 1998 90 606 611 10.1093/jnci/90.8.606 9554443

[B17] Nutting C Camplejohn RS Gilchrist R A patient with 17 primary tumours and a germ line mutation in TP53: tumour induction by adjuvant therapy? Clin Oncol (R Coll Radiol) 2000 12 300 304 11315715 10.1053/clon.2000.9179

[B18] Limacher JM Frebourg T Natarajan-Ame S Two metachronous tumors in the radiotherapy fields of a patient with Li-Fraumeni syndrome Int J Cancer 2001 96 238 242 10.1002/ijc.1021 11474498

[B19] Salmon A Amikam D Sodha N Rapid development of post-radiotherapy sarcoma and breast cancer in a patient with a novel germline 'de-novo' TP53 mutation Clin Oncol (R Coll Radiol) 2007 19 490 493 17572079 10.1016/j.clon.2007.05.001

[B20] Bollet MA Sigal-Zafrani B Mazeau V Age remains the first prognostic factor for loco-regional breast cancer recurrence in young ( < 40 years) women treated with breast conserving surgery first Radiother Oncol 2007 82 272 280 10.1016/j.radonc.2007.01.001 17287037

[B21] Prise en charge du cancer du sein infiltrant de la femme non ménopausée Oncologie 2009 11 507 532 10.1007/s10269-009-1818-6

[B22] Livasy CA Karaca G Nanda R Phenotypic evaluation of the basal-like subtype of invasive breast carcinoma Mod Pathol 2006 19 264 271 10.1038/modpathol.3800528 16341146

[B23] Veronesi U Marubini E Del Vecchio M Local recurrences and distant metastases after conservative breast cancer treatments: partly independent events J Natl Cancer Inst 1995 87 19 27 10.1093/jnci/87.1.19 7666458

[B24] Fowble BL Schultz DJ Overmoyer B The influence of young age on outcome in early stage breast cancer Int J Radiat Oncol Biol Phys 1994 30 23 33 8083119 10.1016/0360-3016(94)90515-0

[B25] Rubino C de Vathaire F Shamsaldin A Radiation dose, chemotherapy, hormonal treatment and risk of second cancer after breast cancer treatment Br J Cancer 2003 89 840 846 10.1038/sj.bjc.6601138 12942115 PMC2394476

[B26] Delia D Goi K Mizutani S Dissociation between cell cycle arrest and apoptosis can occur in Li-Fraumeni cells heterozygous for p53 gene mutations Oncogene 1997 14 2137 2147 10.1038/sj.onc.1201050 9174049

[B27] Mitchel RE Jackson JS Carlisle SM Upper dose thresholds for radiation-induced adaptive response against cancer in high-dose-exposed, cancer-prone, radiation-sensitive Trp53 heterozygous mice Radiat Res 2004 162 20 30 10.1667/RR3190 15222780

[B28] Tinat J Bougeard G Baert-Desurmont S 2009 version of the Chompret criteria for Li Fraumeni syndrome J Clin Oncol 2009 27 e108 109 author reply e110 10.1200/JCO.2009.22.7967 19652052

